# TFDP3 Regulates Epithelial-Mesenchymal Transition in Breast Cancer

**DOI:** 10.1371/journal.pone.0170573

**Published:** 2017-01-23

**Authors:** Kailin Yin, Yanchen Liu, Ming Chu, Yuedan Wang

**Affiliations:** 1 Department of Immunology, School of Basic Medical Science, Peking University, Beijing, China; 2 School of Pharmacy, Liaoning University of Traditional Chinese Medicine, Liaoning, China; University of South Alabama Mitchell Cancer Institute, UNITED STATES

## Abstract

Breast cancer remains a lethal disease to women due to lymph node metastasis, the tumor microenvironment, secondary resistance and other unknown factors. Several important transcription factors involved in this disease, such as PTEN, p53 and beta-catenin, have been identified and researched in-depth as candidates for targeted therapy in breast cancer. TFDP3 is a new, promising candidate for transcriptional regulation in breast cancer, although it was first identified in hepatocellular carcinoma. Here, we demonstrate that TFDP3 is expressed in a variety of malignancies, normal testis tissue and breast cancer cell lines and thus provide evidence that TFDP3 is a cancer-testis antigen. We illustrate that overexpression or silencing TFDP3 interferes with epithelial-mesenchymal transition but does not influence cell proliferation, indicating that the TFDP3 protein acts as a transcription factor during epithelial-mesenchymal transition. These data highlight that TFDP3 is expressed in breast cancer, that it is a member of the cancer-testis antigen family and that it functions as a regulator in epithelial-mesenchymal transition.

## Introduction

Worldwide, breast cancer remains an unsettled medical problem despite the application of drugs or surgeries over the last several decades. Emerging evidence has clarified the principle of individual treatment for breast cancer. For example, less extensive surgery or radiotherapy is recommended for those diagnosed with early breast cancer [1]. The clinical prognosis of breast cancer was linked to the status of the carcinoma and the medical programs used to treat it. The former includes lymph node metastasis, histological grade, the mutation of tumor suppressor genes and amplification of human epidermal growth factor receptor 2 (HER2) and the status of the estrogen and progesterone receptors, while the latter includes chemotherapy, radiotherapy, surgery, hormonal therapy and trastuzumab treatment [2]. Among those factors, HER2 plays an important role in the pathogenesis and prognosis of breast cancer. HER2 is slightly different from the other members of the epidermal growth factor receptor family, and it is defined as an independent prognostic factor for breast cancer and a target for molecular therapy [3,4].

Epithelial-mesenchymal transition (EMT) acts during both physiological and pathological activities. During EMT, epithelial cells lose their polarity and intracellular adhesion forces, but gain the ability to migrate through the basement membrane [5,6]. The physiological process includes embryogenesis and wound healing, while the pathological process consists of tissue fibrosis and tumor metastasis [7]. EMT is of interest because it is regarded as the initial step of distant organ metastasis for carcinomas. During EMT, cells of the solid tumor first obtain the capacity to break through the basic layer of the extracellular matrix and then migrate to other tissues or organs through the blood or lymphatic vessels. Several signaling pathways and transcription factors, such as TGF-beta (signaling pathway) and SNAIL (transcription factor), have been reported to be involved in the EMT process. Several epithelial or mesenchymal markers, such as E-cadherin (epithelial) and N-cadherin (mesenchymal) [8,9], have also been identified.

Human transcription factor dimerization partner family member 3 (TFDP3) was first identified in hepatocellular carcinoma in 2002 by Chen WF, who used a method involving serological analysis of recombinant cDNA expression libraries [10]. TFDP3 was found to dimerize with E2F1 to inhibit the biological function of E2F1, which is involved in p53-dependent or independent cell apoptosis and cell proliferation [11,12]. The negative regulatory effect of TFDP3 on E2F1 was speculated to result from a sequence localized to the C-terminus rather than the DNA binding region [13]. Recently, TFDP3 and some other transcription regulators were found to have a critical role in the gene interaction network in breast cancer [14]. The role of TFDP3 in EMT in breast cancer, however, has not been identified yet.

## Materials and Methods

### Immunohistochemistry

Tissue microarrays were dewaxed by soaking twice in xylene for 10 min. Next, the slide was sequentially immersed in 100%, 95%, 85% and 70% ethanol for 5 min each; immersed in distilled water for 5 min; and then washed twice with PBS for 5 min. The microarray was incubated with 3% H_2_O_2_ at room temperature for 10 min and then washed twice with PBS for 5 min. Antigen retrieval was carried out twice in 0.01 M sodium citrate buffer, and the slide was then cooled at room temperature for approximately 15–30 min before it was washed twice with PBS for 5 min. The microarray was incubated in blocking solution for 20–30 min at room temperature followed by a primary antibody (1:50) at room temperature for 1–2 hours. After the microarray was washed twice with PBS, it was incubated in secondary antibody at room temperature for 15–30 minutes and then washed twice again. The DAB reagent was added at room temperature for 3–5 minutes, and the microarray was washed with distilled water. Finally, hematoxylin was added, and the microarray was washed with distilled water. The microarray was prepared and observed under a microscope.

### Cell culture

The MDA-MB-231 and HCC1954 cell lines were purchased from ATCC. All of the procedures were carried out according to guidelines on the ATCC website. The complete growth media were DMEM (Gibco) containing 10% fetal bovine serum (Gibco) (MDA-MB-231) and RPMI 1640 (Gibco) containing 10% fetal bovine serum (HCC1954).

### CFSE staining

CFSE was purchased from Cayman. All of the procedures were carried out according to the manufacturer’s protocol. The same volume of PBS was added to a CFSE stock solution (10 mg of CFSE diluted into 10 ml of DMSO) to prepare the working solution. The cells were centrifuged at 1250 rpm for 5 min and were resuspended in 5 ml of PBS. Five milliliters of the CFSE working solution was then added to the cell suspension to obtain a final CFSE concentration of 0.25 mg/ml. The cells were incubated at 37°C for 30 min, centrifuged once again at 1250 rpm for 5 min and then resuspended in 10 ml of complete growth medium (10% FBS). One milliliter of the cell suspension was collected for fluorescence detection by flow cytometry every day for 5 to 7 days. All of the procedures were carried out in the dark.

### CCK-8 detection

The CCK-8 detection kit was purchased from ZOMANBIO. We carried out tests according to the manufacturer’s protocol. The cells were seeded into a 96-well plate (100 μl per well), and the cells were pre-cultured at 37°C. Ten microliters of the CCK solution was added into each well, and the plate was then shaken gently. The plate was incubated at 37°C for 1–4 hours, and the OD 450 was measured with a microplate reader (Thermo).

### Transfection

All of the procedures were carried out according to the manufacturer’s protocol. The cells were seeded the day before transfection so that they would be 70–90% confluent at the time of transfection. The Lipofectamine 3000 reagent (Invitrogen) was diluted into Opti-MEM medium (Gibco), mixed and then placed at room temperature for 5 min. DNA for transfection was diluted into the same amount of Opti-MEM medium, mixed and then placed at room temperature for 5 min. Diluted DNA and diluted Lipofectamine 3000 reagent were mixed and then incubated at room temperature for 15–30 min. The complex was added to the cells in 6-well plates, and the cells were incubated for 24–72 hours at 37°C. The transfected cells were then analyzed and used for subsequent experiments.

### Wound healing assay

The wound healing assay was carried out when the cells reached 80–90% confluence in a 24-well plate (Corning). Utilizing a small pipette tip (Axygen), a line was drawn on the bottom of the well, and photos of the scratch were then taken under a microscope (Olympus). The wound healing was observed and photographed every 24 hours. The cell number located on the scratch was counted, and the area of the remaining scratch site was calculated. The observation continued until the cells reached 100% confluence at the scratch site and the wound area disappeared under the microscope.

### Transwell assay

Cells were centrifuged and resuspended in base medium 1640 (Gibco) or DMEM (Gibco) without fetal bovine serum. A 200-μl cell suspension was added onto each chamber of the transwell plate (24-well plate, 12 chamber, aperture of 8.0 μm) (Corning), and 1000 μl of complete medium (10% FBS) was added on the bottom of the well (below the chamber). The chamber was placed above the complete medium but within the well and was then incubated at 37°C for 24–72 hours.

The chamber was removed from the plate after incubation and was washed three times with PBS at room temperature. The cells on the upper surface of the chamber were wiped out with cotton swabs, and the chamber was then washed with PBS. The cells on the lower surface of the chamber were fixed with 4% paraformaldehyde for 30 min at room temperature and then washed. The cells were stained with 0.1% crystal violet for 20 min at room temperature and then washed. The chamber was dried by placing it upside down overnight at room temperature. Five evenly distributed vision fields on the lower surface of the chamber were selected and photographed under the microscope (Olympus).

### Clonogenic assay

Three-hundred cells were seeded into each well of a 6-well plate (Corning) containing complete growth medium and were then incubated at 37°C for 14 days. The cell suspension was pipetted repeatedly, and the plate was shaken gently to ensure a single-cell suspension. The cells were fixed with 4% paraformaldehyde and then stained with 0.1% crystal violet. The complete vision field of each well was examined under a microscope.

### Immunofluorescence

Cells were washed three times with PBS and were then fixed with 4% pre-cooled paraformaldehyde for 15 min. The cells were washed with PBS and then permeabilized with 0.1% triton-X100 (diluted in PBS) for 20 min at room temperature. The cells were washed with PBS (containing 0.1% triton) as described above and were then incubated with 5% bovine serum albumin (BSA) for 30 min at room temperature. The coverslips were dried with absorbent paper and incubated with primary antibody overnight at 4°C. The cells were washed with PBS (containing 0.1% triton) as stated above, incubated with a fluorescence-conjugated secondary antibody at 37°C for 1 h in the dark and then washed. The cells were incubated with DAPI for 90 seconds, washed with PBS and then prepared for observation under a fluorescence microscope (Olympus).

### Flow cytometry

Samples were prepared for detection in a flow cytometer (BD, C6), and all of the procedures were carried out according to the manufacturer’s protocol. The samples were carefully shaken before loading to avoid cell clusters. The speed for loading the cells was slow, and 10000 cells were collected from each tube. The samples were loaded into the flow cytometer as soon as possible after the preparation procedure. Some of the samples were kept in the dark to avoid fluorescence quenching.

### RNA extraction

The Total RNA Kit was purchased from OMEGA, and the RevertAid First Strand cDNA Synthesis Kit was purchased from Thermo. RNA extraction and reverse transcription were carried out according to the manufacturer’s protocol. Cells were centrifuged at 1500 rpm for 5 min and then washed three times with PBS. Three-hundred-fifty microliters of TRK lysis buffer was added to a maximum of 5x10^6^ cells, and 600 μl of TRK lysis buffer was added to a maximum of 1x10^7^ cells. The cell lysis solution was homogenized by vortexing for 1 min and centrifuged at 12000 rpm for 10 min. The supernatant was transferred to a Mini Column, and the same amount of 70% ethanol (diluted in DEPC water) was added. The Mini Column was then transferred to a 2-ml collection tube and centrifuged at 12000 rpm for 1 min. The pellet was sequentially washed once with 300 μl of RNA Wash Buffer I, once with 500 μl of RNA Wash Buffer I and twice with 500 μl of RNA Wash Buffer II; the column was centrifuged at each step. The Mini Column was centrifuged at 12000 rpm for 2 min to remove extra liquid. Thirty to fifty microliters DEPC water was added to the Mini Column, and the eluate was collected in another RNase-free EP tube. The purity and concentration of RNA were determined for subsequent experiments. All of the procedures were carried out at room temperature unless otherwise stated.

### Real-time PCR

TransStart Tip Green qPCR SupreMix was purchased from TransGen Biotech, and all of the procedures were carried out according to the manufacturer’s protocol. For TFDP3, the forward primer was 5’-ATGGACGAGAACCAGACCAG-3’ and the reverse primer was 5’-CCCAGACCTTCATGGAAAGA-3’. For GAPDH, the forward primer was 5’-AATGACCCCTTCATTGAC-3’ and the reverse primer was 5’-TCCACGACGTACTCAGCGC-3’. The cycling parameters were 94°C for 15 s, 60°C for 30 s and 72°C for 60 s; 40 cycles were carried out.

### Tissue microarray

Tissue microarrays FDA800, BR486 and BR963a were purchased from Alenabio, and HBre-Duc150Sur-02 was purchased from Shanghai Outdo Biotech Company. Detailed information on tissue microarrays FDA800, BR486 and BR963a are available at http://www.alenabio.com/tissue-arrays/All/, and information on HBre-Duc150Sur-02 is available at http://www.superchip.com.cn/product/detail_234.aspx.

### Statistics

All of the data in this paper were analyzed with SPSS 20. Student’s t-test and a Chi-square test were carried out to compare the significant differences. *p<0.05, **p<0.01 and ***p<0.001; NS represents no significant differences. Survival analysis was performed using the Kaplan-Meier method, and Log Rank was used for statistical analysis.

## Results

### TFDP3 is expressed in testis and malignancies

**Fig 1:**
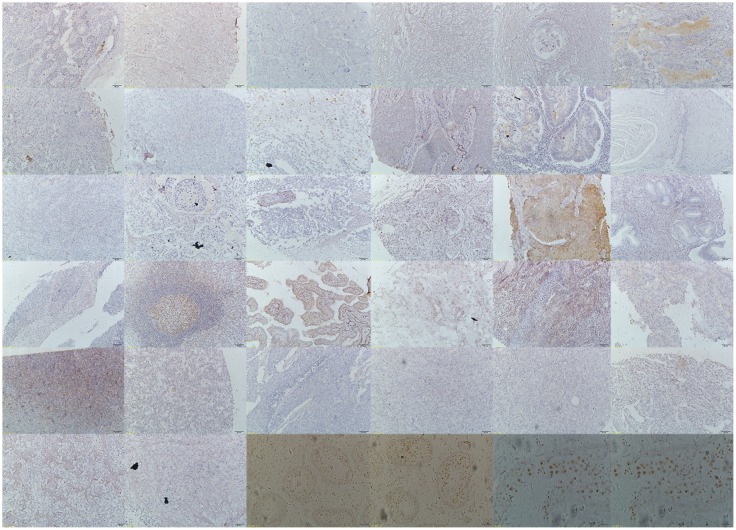
Expression profiles of TFDP3 in multi-malignancies as detected by tissue microarray. The first 32 immunohistochemistry photos belong to tissue microarray FDA800. They are the skin, lung, testis, prostate, gallbladder, small intestine, pancreas, colon, rectum, esophagus, vermiform appendix, tongue, parotid gland, stomach, liver, breast, uterus, cervix, ovary, mediastinum, thyroid, brain, bladder, kidney, lymph node, soft tissue, retroperitoneum, abdominal wall, abdominal cavity, pelvic cavity, bone and peritoneum. The last four photos are from normal testis tissue. Photos 33 and 34 are 10*. Photos 35 and 36 are 40*. The first row contains photos 1 to 6, and so forth.

It has been reported that TFDP3 is a cancer-testis antigen [10]. Therefore, we determined the expression status of TFDP3 in normal testis tissue by immunohistochemistry and found that TFDP3 was present in testis tissue (Fig 1). Next, we used the tissue microarray FDA800 to detect the expression level of TFDP3 in multi-malignancies. The results of immunohistochemistry showed that TFDP3 was present in the malignancies of multiple organs, including the skin, lung, prostate, gallbladder, small intestine, pancreas, rectum, esophagus, vermiform appendix, stomach, breast, uterus, ovary, mediastinum, thyroid, brain, bladder, kidney, lymph node, soft tissue and pelvic cavity (Fig 1). These data provided evidence that TFDP3 is a cancer-testis antigen and proved that TFDP3 is widely expressed in malignancies at the protein level.

### TFDP3 is expressed in breast cancer tissues

**Fig 2:**
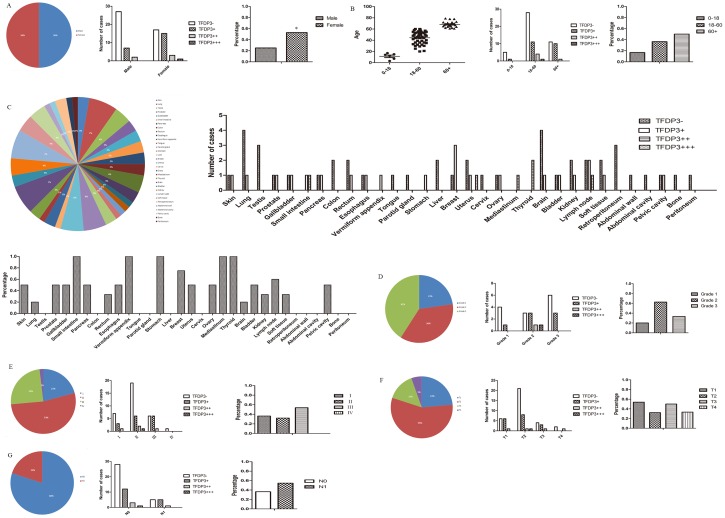
Statistical analysis of TFDP3 in tissue microarray FDA800. (A-B) TFDP3 expression in sex and age groups. (C) TFDP3 expression profiles in multi-malignancies. (D-E) TFDP3 expression in samples of different clinical and pathological grades. (F-G) TFDP3 expression in the T and N stages.

Tissue microarray FDA800 contains information on different carcinomas from 72 samples, and it was of interest because information regarding age, sex, and TNM stage was provided for each case. The following malignancies were negative for TFDP3 expression: testis, colon, tongue, parotid gland, liver, cervix, retroperitoneum, abdominal wall, abdominal cavity, bone and peritoneum (Figs 1 and 2C). The cut-off for age was 18 and 60 since there were both males and females in the group; the cut-off was a rough dividing line for children, adults and the elderly (Fig 2B). We found that there were significant TFDP3 staining differences between the sexes (p = 0.02) (Fig 2A), but there were no significant differences with regard to age (p = 0.285) (Fig 2B), clinical grade (p = 0.264) (Fig 2D), clinical stage (p = 0.489) (Fig 2E), T stage (p = 0.532) (Fig 2F) or N stage (p = 0.271) (Fig 2G). As for sex (p = 0.02), the rate of TFDP3 expression was 25% in the male group (9 in 36 cases) and 52.78% in the female group (19 in 36 cases) (Fig 2A). The results suggested that TFDP3 is highly expressed in females, which might be related to estrogen and progesterone in women. As for age (p = 0.285), the rate of TFDP3 expression was 16.67% in the lower age group (1 in 6 cases), 36.37% in the middle age group (16 in 44 cases) and 50% in the higher age group (11 in 22 cases) (Fig 2B). These findings suggest that TFDP3 expression might be positively correlated with age, although there were no significant differences in this study.

**Fig 3:**
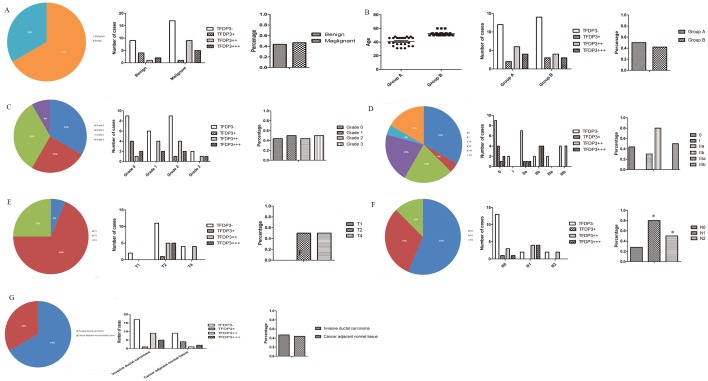
Statistical analysis of TFDP3 expression in tissue microarray BR486. (A-B) TFDP3 expression in different pathological types and age groups. (C-D) TFDP3 expression in samples of different pathological grades and clinical stages. (E-F) TFDP3 expression in the T and N stages. (G) TFDP3 expression in samples of different pathological diagnoses.

Tissue microarray BR486 contained invasive breast ductal carcinoma and the matching edge tissue for 48 cases. A pathological diagnosis of grade 1–4 was indicated as well-differentiated, moderately differentiated, poorly differentiated or undifferentiated. The cut-off for the age group was 49 since menopause occurs at this age and all of the cases in the microarray were female (this cut-off was also applied to other tissue microarrays unless otherwise noted). The pathological diagnosis consisted of invasive ductal carcinoma and cancer-adjacent normal tissue. The rate of TFDP3 expression was 46.88% in all of the invasive ductal carcinoma cases, indicating that TFDP3 is present in clinical breast cancer samples (Fig 3G). We found that there were significant differences in the expression level of TFDP3 in terms of the N stage (p = 0.029) (Fig 3F), but there were no significant differences in the expression level of TFDP3 with regard to the pathological types (p = 0.838) (Fig 3A), age (p = 0.562) (Fig 3B), pathological grade (p = 0.983) (Fig 3C), clinical stage (p = 0.102) (Fig 3D), T stage (p = 0.390) (Fig 3E) or pathological diagnosis (p = 0.838) (Fig 3G). As for age (p = 0.562) (cut-off of 49), the results implied that changes in endocrine hormone levels might not interfere with TFDP3 expression (Fig 3B). As for the N stage (p = 0.029), the rate of TFDP3 expression in the N0 stage was 27.78% (5 in 18 cases) and 71.43% in the non-N0 stage (10 in 14 cases) (Fig 3F), indicating that the overexpression of TFDP3 might contribute to lymph node metastasis, thus providing a link between TFDP3 expression and tumor metastasis in breast cancer.

**Fig 4:**
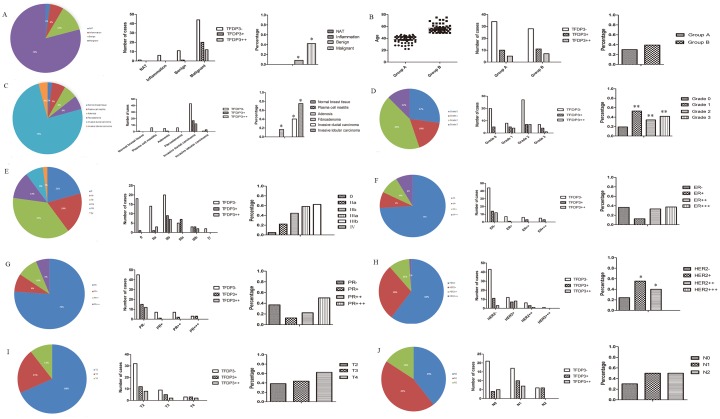
Statistical analysis of TFDP3 in tissue microarray BR963a. (A-B) TFDP3 expression in different pathological types and age groups. (C-E) TFDP3 expression in samples of different pathological diagnoses, clinical grades and clinical stages. (F-H) TFDP3 expression in the ER, PR and HER2 groups. (I-J) TFDP3 expression in the T and N stages.

Tissue microarray BR963a contained 96 cases of multiple types of breast diseases, namely, normal breast tissue, plasma cell mastitis, adenosis, fibroadenoma, invasive ductal carcinoma and invasive lobular carcinoma. BR963a also included information on the ER/PR/HER2 staining results, TNM, clinical stage and pathological stage of the patients. Our data showed that there were significant TFDP3 staining differences with regard to the pathological diagnosis (p = 0.04) (Fig 4A), pathological type (p = 0.026) (Fig 4C), pathological grade (p = 0.005) (Fig 4D) and HER2 staining (p = 0.038) (Fig 4H), but there were no significant differences in TFDP3 staining with regard to age (p = 0.347) (Fig 4B), clinical stage (p = 0.162) (Fig 4E), ER staining (p = 0.581) (Fig 4F), PR staining (p = 0.358) (Fig 4G), T stage (p = 0.435) (Fig 4I) and N stage (p = 0.225) (Fig 4J). As for the pathological diagnosis (p = 0.04), the rate of TFDP3 expression was 75% in invasive lobular carcinoma (3 in 4 cases), 40.28% in invasive ductal carcinoma (29 in 72 cases) and 16.67% in adenosis (1 in 6 cases), while there was no TFDP3 staining detected in fibroadenoma (0 in 6 cases), plasma cell mastitis (0 in 6 cases) or normal breast tissue (0 in 2 cases) (Fig 4A). As for the pathological types (p = 0.026), the rate of TFDP3 expression was 42.11% in malignant disease (32 in 76 cases) and 8.33% in benign tissue (1 in 8 cases), while no TFDP3 staining was seen in the inflammation (0 in 6 cases) and NAT groups (0 in 2 cases) (Fig 4C). Collectively, these data illustrated that TFDP3 might be exclusively expressed in carcinomas since it was not expressed in inflammatory diseases or in the benign tissues of the breast. As for the pathological grade (p = 0.005), the rate of TFDP3 expression was 19.2% in grade 0 (5 in 26 cases), 52.9% in grade 1 (9 in 17 cases), 34.1% in grade 2 (14 in 41 cases) and 41.7% in grade 3 (5 in 12 cases) (Fig 4D). These results support a correlation between TFDP3 and the degree of breast cancer. As for HER2 staining (p = 0.038), the rate of TFDP3 expression was 24.14% in the HER2-negative group (14 in 58 cases) and 50% in the HER2-positive group (19 in 38 cases) (Fig 4H), indicating that HER2 amplification might lead to TFDP3 activation in breast cancer. As for the clinical stage (p = 0.162), the rate of TFDP3 expression was 5% in stage 0 (1 in 20 cases), 37.04% in stage II (20 in 54 cases) and 60% in stage III (12 in 20 cases), but TFDP3 was not expressed in stage IV (0 in 2 cases) (Fig 4E). The data revealed that the overexpression of TFDP3 might be related to the progression or metastasis of breast cancer, although no significant differences were detected in this study.

**Fig 5:**
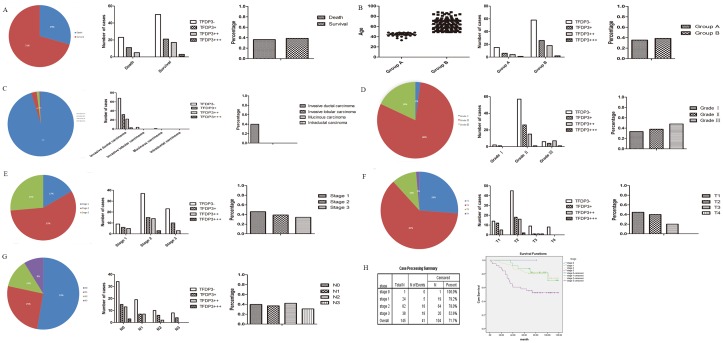
Statistical analysis of TFDP3 in tissue microarray HBre-Duc150Sur-02. (A) Correlation between the TFDP3 expression level and survival rates. (B-C) TFDP3 expression in different age groups and histological types. (D-E) TFDP3 expression in samples of different pathological grades and clinical stages. (F-G) TFDP3 expression in the T and N stages. (H) Kaplan-Meier method for survival analysis.

Tissue microarray HBre-Duc150Sur-02 contained information about the survival time of 150 individuals diagnosed with breast cancer. Surgery was performed between 2004.8 and 2008.12, and the follow-up period, which lasted for 5.6 to 10 years, ended in 2014.7. We analyzed the data using the Kaplan-Meier test and found that there were significant differences between the clinical stages (0–3) and the survival rates (p = 0.007) (Fig 5H). There were no significant TFDP3 staining differences with regard to the survival rates (p = 0.671) (Fig 5A), age (p = 0.46) (Fig 5B), histological type (p = 0.131) (Fig 5C), pathological grade (p = 0.161) (Fig 5D), clinical stage (p = 0.366) (Fig 5E), T stage (p = 0.188) (Fig 5F) and N stage (p = 0.818) (Fig 5G). In terms of survival rates (p = 0.671), the data revealed that the expression level of TFDP3 might not affect the clinical outcome of breast cancer or that TFDP3 might not be an independent prognostic factor for breast cancer. As for the histological types (p = 0.131), we found that the rate of TFDP3 expression was 39.86% in invasive ductal carcinoma (57 in 143 cases), but that TFDP3 was not expressed in invasive lobular carcinoma (0 in 4 cases), mucinous carcinoma (0 in 2 cases) or intraductal carcinoma (0 in 1 cases) (Fig 5C). These results might suggest a specific TFDP3 expression pattern in different types of breast carcinomas, although no significant differences were found in this study.

### TFDP3 is expressed in breast cancer cell lines

**Fig 6:**
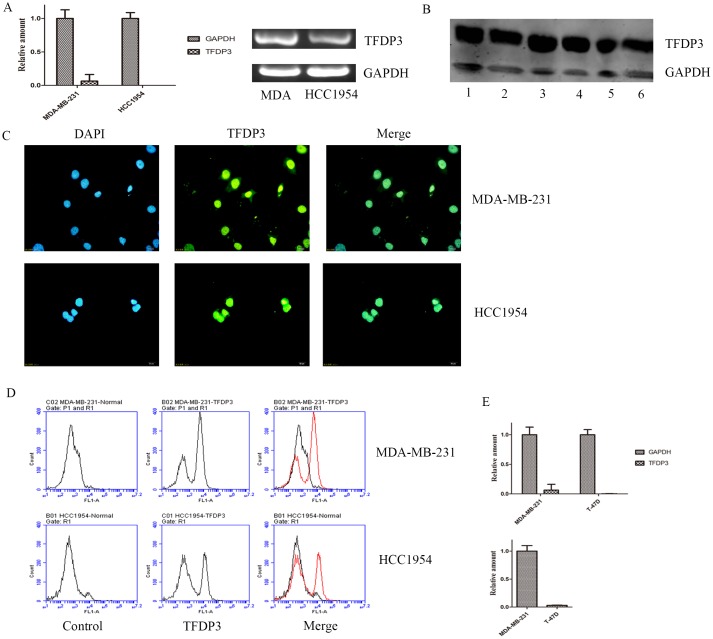
Expression status of TFDP3 in breast cancer cell lines. (A) TFDP3 expression at the mRNA level. (B) TFDP3 expression at the protein level. Lanes 1, 3 and 5 represent MDA-MB-231; and lanes 2, 4 and 6 represent HCC1954. Lanes 1 and 2 contained total protein, lanes 3 and 4 contained cytoplasmic proteins and lanes 5 and 6 contained nuclear proteins. (C) TFDP3 expression at the protein level as determined by immunofluorescence. (D) TFDP3 expression at the protein level as determined by flow cytometry. (E) TFDP3 expression in MDA-MB-231 and T-47D cell lines.

Since immunohistochemistry revealed that TFDP3 is expressed in breast cancer tissues, we sought to determine the expression level of TFDP3 in breast cancer cell lines. TFDP3 was expressed in the MDA-MB-231 (adenocarcinoma) and HCC1954 (ductal carcinoma) cell lines, both at the mRNA and protein levels (Fig 6A and 6B). To further confirm our speculation that TFDP3 is expressed in breast cancer cell lines, we carried out immunofluorescence and flow cytometry. Cells were stained with green fluorescence, showing that TFDP3 is present in MDA-MB-231 and HCC1954. We also observed positive staining forTFDP3 in both the cytoplasm and the nucleus, indicating that the TFDP3 protein might be located in the nucleus and cytoplasm (Fig 6C). A new peak was detected by flow cytometry in the FL1 channel after antibody staining, further confirming that TFDP3 is expressed in breast cancer cell lines (Fig 6D). Collectively, these data suggest that TFDP3 is expressed in breast cancer cell lines.

To further determine the expression status of TFDP3 in breast cancer cell lines, we selected MDA-MB-231 (basal type of breast cancer, mesenchymal phenotype) and T-47D (luminal type of breast cancer) cell lines. Our data showed that TFDP3 was highly expressed in mesenchymal cell line but less expressed in luminal type of breast cancer (Fig 6E). Our data also showed that relative amount of TFDP3 in MDA-MB-231 was higher than that in T-47D (Fig 6E). These results proved that TFDP3 is preferentially expressed in mesenchymal while less expressed in luminal type of breast cancer.

### TFDP3 regulates epithelial-mesenchymal transition

**Fig 7:**
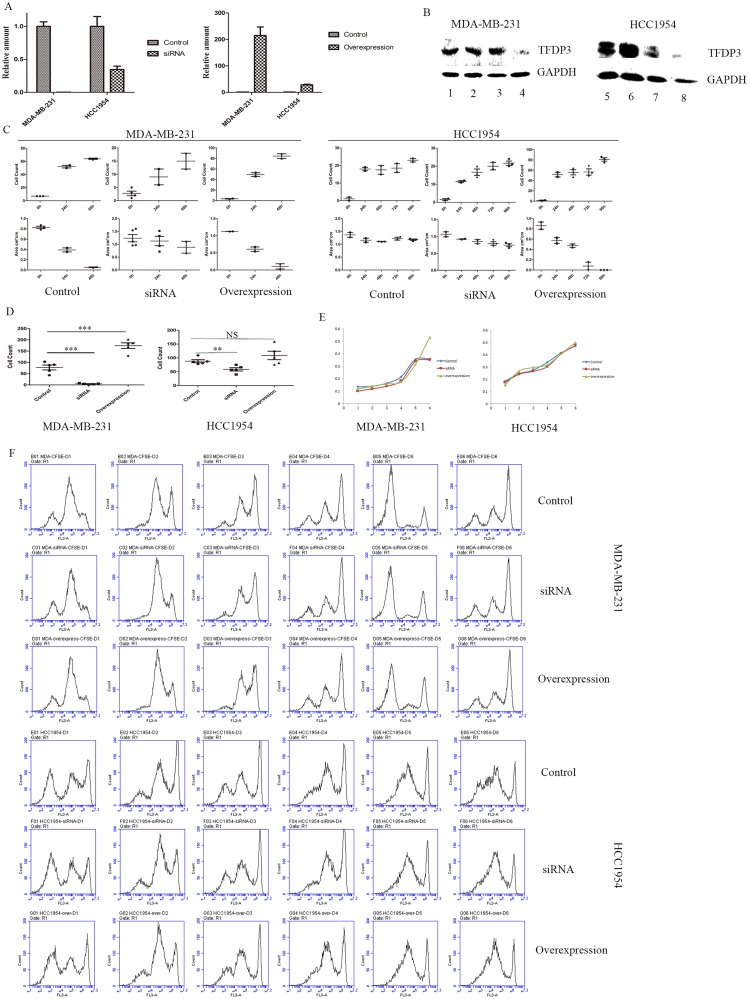
Role of TFDP3 in epithelial-mesenchymal transition. (A) TFDP3 expression at the mRNA level after the transfection of siRNA or an overexpression plasmid. (B) TFDP3 expression at the protein level after transfection. Lanes 1, 2, 3 and 4 represent MDA-MB-231; and lanes 5, 6, 7 and 8 represent HCC1954. Lanes 1 and 5 contained the control, lanes 2 and 6 contained the overexpression group, lanes 3 and 7 contained the empty vector group and lanes 4 and 8 contained the siRNA group. (C) The results of the wound healing assay from day 1 to day 3 (MDA-MB-231) or from day 1 to day 5 (HCC1954). (D) Cell count in the transwell experiment in the control, siRNA and overexpression groups. (E) CCK-8 test for cell proliferation rates in the control, siRNA and overexpression groups. (F) CFSE results from day 1 to day 6 in the control, siRNA and overexpression groups.

Because TFDP3 was correlated with the clinical N stage, we asked whether TFDP3 expression was correlated with the EMT phenotype in breast cancer cells. We constructed two sister cell lines using a TFDP3-overexpression plasmid and the TFDP3 siRNA sequence. TFDP3 was downregulated after siRNA transfection and upregulated after the transfection of the overexpression plasmid, confirming that TFDP3 was silenced and overexpressed, respectively, at the mRNA level (Fig 7A). Additionally, the TFDP3 protein levels were reduced in the siRNA group, indicating that TFDP3 was silenced at the protein level (Fig 7B). Interestingly, the TFDP3 protein levels remained unchanged after transfection of the overexpression plasmid into the MDA-MB-231 cell line (Fig 7B). This might be due to high background levels.

A wound healing assay was performed to compare the differences in the wound healing ability of cells undergoing EMT among the overexpression, siRNA and control groups. The number of cells on the scratch site and remaining wound area were measured to evaluate the state of wound healing. Forty-eight hours after scratching, we observed that the scratch site was almost completely occupied by cells in the control and overexpression groups, while no significant changes were seen in the siRNA group. The rate of wound healing was high in the control and overexpression groups, but was low the in siRNA group (Fig 7C). We analyzed the microscopy data and concluded that the sum of cells was very high in the overexpression group, high in the control group and low in the siRNA group (Fig 7C). Similar results were observed in the HCC1954 cell line (Fig 7C). A transwell experiment was subsequently carried out to confirm that TFDP3 is involved in EMT. Cells that passed through the basement membrane were measured as a standard for transwell efficiency. Our data showed that the cell number was normal in the control group, high in the overexpression group and low in the siRNA group (Fig 7D). These data suggest that the overexpression of TFDP3 would accelerate the progression of EMT, while the silencing of TFDP3 would inhibit EMT.

Since TFDP3 abrogates G1 to S phase progression by inhibiting the binding of E2F1 to DNA [12], we asked whether the effect of TFDP3 on EMT would interfere with cell proliferation. We analyzed the cell growth curves from a CCK-8 experiment and found that there were no significant differences in the growth rates among the three groups from day 1 to day 5 (Fig 7E). Interestingly, the proliferation of the MDA-MB-231 cells in the overexpression group was higher than that of the other two groups on day 6 (Fig 7E). Additionally, we carried out CFSE staining to compare the differences in cell proliferation. We observed that the height and number of CFSE peaks remained the same among the three groups each day, suggesting that the expression level of TFDP3 was not involved in the modulation of cell proliferation (Fig 7F). The results of a clonogenic assay showed that there were 10 newly formed MDA-MB-231 colonies from 300 primary cells in the control group, 9 in the siRNA group and 8 in the overexpression group, while there were 2 newly formed HCC1954 colonies in the control group, 3 in the siRNA group and 2 in the overexpression group. Our data indicate that the silencing or overexpression of TFDP3 would not interfere with cell proliferation.

**Fig 8:**
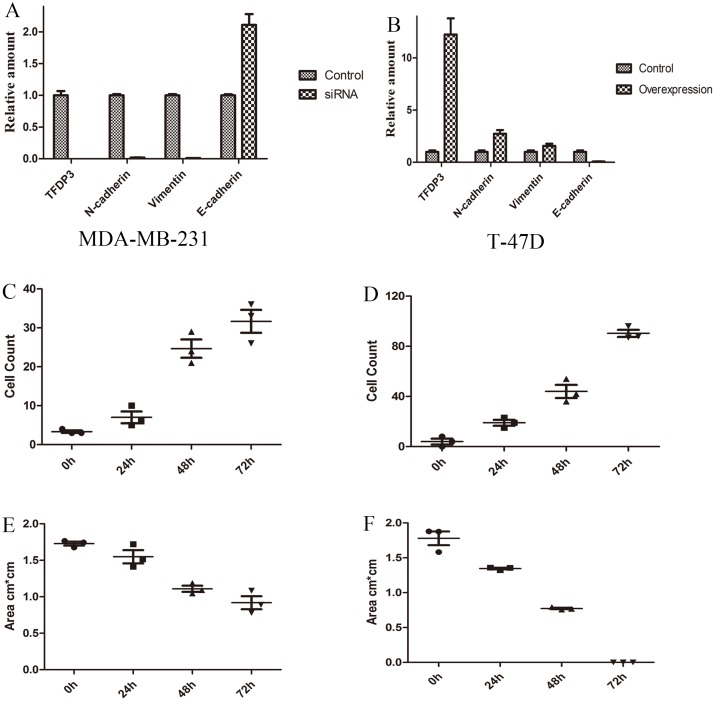
TFDP3 regulates the expression of EMT-related markers. (A) Expression of EMT-related markers in MDA-MB-231 cell line after siRNA treatment. (B) Expression of EMT-related markers in T-47D cell line after overexpression plasmid transfection. (C) Cell count in wound healing assay in control group (T-47D). (D) Cell count in wound healing assay in overexpression group (T-47D). (E) Wound area in wound healing assay in control group (T-47D). (F) Wound area in wound healing assay in overexpression group (T-47D).

We performed a knockdown experiment of TFDP3 in mesenchymal type of breast cancer (MDA-MB-231) utilizing siRNA, and our data showed that TFDP3 was knocked down after siRNA transfection (relative amount 0.0012 vs 1.0) (Fig 8A). The data also showed that N-cadherin (mesenchymal marker) (relative amount 0.019 vs 1.0) and Vimentin (mesenchymal marker) (relative amount 0.010 vs 1.0) were downregulated after knockdown of TFDP3, while E-cadherin (luminal marker) were upregulated to 2.11 fold after knockdown of TFDP3 (Fig 8A). These results proved that knockdown of TFDP3 in MDA-MB-231 would decrease mesenchymal marker and correspondingly increase luminal marker expression. Our data also showed that cell count in siRNA group was lower than that in control group (Fig 7C). The remaining wound area decreased in control group while remained unchanged in siRNA group (Fig 7C). These results provided evidence that reduced expression of TFDP3 would impair their migratory and invasive activity. Taken together, we proved that knockdown of TFDP3 in mesenchymal types of breast cancer would decrease mesenchymal markers while increase luminal markers, and this process would affect the migration capacity of cells.

We performed an overexpression experiment of TFDP3 in luminal type of breast cancer (T-47D) utilizing overexpression plasmid, and our data showed that the expression level of TFDP3 was upregulated to 12.2 fold after overexpression plasmid transfection (Fig 8B). We also reported that N-cadherin (mesenchymal marker) and Vimentin (mesenchymal marker) were upregulated to 2.74 fold (N-cadherin) and 1.57 fold (Vimentin) as compared to control group (Fig 8B). We showed that E-cadherin (luminal marker) was downregulated in overexpression group compared with control group (relative amount 0.076 vs 1.0) (Fig 8B). These results suggested that induced expression of TFDP3 in T-47D cell line would increase mesenchymal markers while correspondingly decrease luminal marker. We also showed that cell count in wound site was high in overexpression group while low in control group (Fig 8C and 8D). At the same time, we found that the remaining wound area was lower in overexpression group than that in control group from 0 h to 72 h (Fig 8E and 8F). The wound area was completely occupied by cells in overexpression group while remained unchanged in control group at 72h (Fig 8E and 8F). Taken together, our data suggested that induced expression of TFDP3 in luminal breast cancer cells (T-47D) would increase mesenchymal markers while correspondingly decrease luminal markers, and this process significantly increases their migratory and invasive activity.

## Discussion

Cancer-testis antigens have two characteristics—the expression spectrum consists of normal testis tissue and multiple types of malignancies, and the genes encoding cancer-testis antigens are localized on X chromosome [15]. Emerging evidence has put forward the notion that cancer-testis antigens should be categorized into three types, namely, testis restricted, testis-brain restricted and testis selective [16], since the testis and brain are both immunological-privileged organs. Previous studies have reported that TFDP3 is expressed in the testis [10] and in multiple cancer tissues, including, but not limited to, the cerebrum, esophagus and skin [17], and that it is weakly expressed in the normal pancreas [10]. In this paper, we found that TFDP3 is expressed in normal testis tissue and in multi-malignancies. The TFDP3 gene mapped to Xq26.2, illustrating that TFDP3 is a cancer-testis antigen. Cancer-testis antigens are ideal candidates for immunotherapy [18] because they are more specific and less toxic than chemotherapy agents [19]. The rate of TFDP3 expression was 46.88% in invasive ductal carcinoma (BR486), providing a platform for targeted therapy for breast cancer. A cancer-testis antigen-based vaccine, rather than traditional molecular targeted therapy, should also be applied to multi-malignancies [18]. Thus, TFDP3 could be a potential target for vaccine development. Apart from their roles in vaccines, cancer-testis antigens are also involved in immune escape, tumor invasion and metastasis [20], which are characteristics of tumorigenesis. However, the relationship between TFDP3 and tumorigenesis has not been identified yet.

Overexpression of HER2 is often related to a poor clinical prognosis [21], but a combination therapy of trastuzumab and lapatinib (tyrosine kinase inhibitor) was reported to have good efficacy on HER2-positive breast cancer [22]. As for metastatic HER2-positive breast cancer, a double-blind, phase 3 trial proved that the combination of pertuzumab, trastuzumab and docetaxel prolonged the survival period compared with the control group (placebo, trastuzumab and docetaxel) [23]. As for HER2-negative but estrogen receptor-positive breast cancer, palbociclib and letrozole were proven to be effective in a phase 2 study [24]. Another study found that trastuzumab emtansine alone could extend the cancer-free survival period in HER2-positive breast cancer patients compared with lapatinib plus capecitabine [25]. Here, we found that there were significant differences between TFDP3 expression and HER2 staining (p = 0.038), indicating that TFDP3 might be involved in the HER2 signaling pathway or that TFDP3 might participate in trastuzumab resistance. A combination therapy targeting TFDP3 as well as HER2 might become an effective method to treat HER2-positive and TFDP3-positive metastatic breast cancer.

Our data revealed that TFDP3 is expressed in breast cancer tissue and breast cancer cell lines. This is the first time that TFDP3 was reported to be expressed in breast cancer, although TFDP3 was reported to be present in U2OS, HEK293, H1288, MCF-7 and HeLa cell lines [13]. The question whether TFDP3 is expressed in other breast cancer cell lines or in normal breast cell lines, however, was still unanswered. Since TFDP3 represses the transcriptional activity of downstream genes by preventing the E2F1 heterodimer from entering the nucleus [26], we speculated that the expression of TFDP3 in breast cancer would cause transcriptional repression of certain genes downstream of E2F1. However, this was not addressed in this paper. The regulatory network involving TFDP3 and E2F1 has not been completely identified yet, and further evidence is needed to clarify the signaling pathway involving TFDP3.

In this paper, we reported that overexpression of TFDP3 accelerates wound healing, while silencing of TFDP3 functions in the opposite direction, suggesting that TFDP3 might be involved in EMT. However, the molecular mechanism remains unclear. Additionally, we showed that overexpression or silencing of TFDP3 does not influence cell proliferation, indicating that TFDP3 might not be involved in cell cycle regulation. This is in contrast to the findings of Chen WF, who showed that expression of TFDP3 leads to G1 to S phase arrest [12]. One explanation for this contradiction is that cell cycle progression was transiently aborted by the exogenous expression of TFDP3. The tumor cells would then undergo normal cell proliferation once TFDP3 was degraded or cleaved by intrinsic pathways, and therefore, there would be a G1 to S phase arrest, but no interference on cell cycle progression. EMT was reported to be reactivated during tumor invasion and metastasis [27]. Therefore, a number of transcriptional regulators, such as Wnt, Notch and TGF-beta signaling molecules [28], which are involved in EMT, are regarded to be promoters of metastasis [29]. TFDP3 was found to participate in the EMT process, as proven by the overexpression and silencing experiments performed on breast cancer cell lines. Therefore, it was necessary to highlight the role of TFDP3 in breast cancer since TFDP3 suppresses gene expression independent of the retinoblastoma tumor suppressor protein pathway [30]. Expression of N-cadherin, Snail, Slug and Twist, and so on increases, while expression of E-cadherin and Occludin decreases during EMT [31]. Thus, the detection and analysis of these EMT markers would help achieve a better understanding of TFDP3 function in EMT. The cytoskeleton of tumor cells is thought to be reorganized to enable cells to pass through the extracellular matrix [32]. Whether TFDP3 is involved in this process is unknown. It has also been reported that highly tumorigenic breast cancer stem cells (CD44^+^CD24^-/low^) are generated through activation of the Ras pathway in EMT [33], providing a direct link between EMT and cancer stem cells. However, whether there is a correlation between TFDP3 and cancer stem cells remains unknown. Collectively, our data provide evidence that TFDP3 is expressed in breast cancer, that it is a member of the cancer-testis antigen family and that it is involved in epithelial-mesenchymal transition.
